# Bacteria and Boar Semen Storage: Progress and Challenges

**DOI:** 10.3390/antibiotics11121796

**Published:** 2022-12-10

**Authors:** María José Contreras, Kattia Núñez-Montero, Pablo Bruna, Matías García, Karla Leal, Leticia Barrientos, Helga Weber

**Affiliations:** 1 Extreme Environments Biotechnology Lab, Center of Excellence in Translational Medicine, Universidad de La Frontera, Av. Alemania 0458, Temuco 01145, Chile; 2 Facultad de Ciencias Agropecuarias y Medioambiente, Universidad de La Frontera, Avenida Francisco Salazar, Temuco 01145, Chile; 3 Biotechnology Research Center, Department of Biology, Instituto Tecnológico de Costa Rica, Cartago 30101, Costa Rica; 4 Scientific and Technological Bioresource Nucleus (BIOREN), Universidad de La Frontera, Temuco 01145, Chile; 5 Center of Excellence in Traslational Medicine (CEMT), Universidad de La Frontera, Av. Alemania 0458, Temuco 01145, Chile

**Keywords:** antibiotics, antibiotic resistance, bacteria contamination, bacteriospermia, boar semen, sperm preservation

## Abstract

Porcine breeding today is based on artificial insemination with chilled semen. This is stored at 5 °C with antibiotic supplementation to avoid bacteriospermia. There are many negative consequences on sperm quality and functionality as a result of bacterial contamination, as well as on the health of the sow. Nowadays, various techniques are being developed to reduce the indiscriminate use of antibiotics and thus avoid the generation of antibiotic resistance genes. This review aims to inform about the bacterial contamination consequences of storing liquid semen from boar and to provide an update on current methods and alternatives to antibiotic use in cold storage.

## 1. Introduction

Artificial insemination (AI) is a widely used technique in swine production. Advances in the technique have made it possible to store spermatozoa at temperatures of 15–20 °C for short periods, up to ten days. Unfortunately, it is currently associated with bacterial contamination of semen during collection and dilution [1,2,3]. Although the temperature is reduced to induce sperm inactivity during storage, bacterial growth can still occur [4]. Bacterial growth has been associated with deleterious effects on semen quality and shelf life, such as sperm agglutination, decreased sperm motility and viability [5,6]. In addition, reproductive output after AI can also be affected by bacteriospermia [6].

Bacterial contamination and its effects on the loss of sperm quality and the transmission of potential pathogens to the inseminated must be prevented to avoid adverse effects on fertility and litter size [1]. That is why antibiotics, such as gentamicin sulfate, are routinely added to AI semen doses according to legal requirements (Council Directive, European Union, 20/686). Around 12.8 million liters of semen containing antibiotics are used worldwide every year [7]. Therefore, large quantities of antibiotics are used prophylactically. Unfortunately, there are more and more resistant bacteria, a high-risk factor for pig production and human health. In particular, important amounts of antibiotics have been reported in liquid manure in pig breeding [7].

The aim of this review is to provide information about the bacterial contamination consequences on the storage of liquid semen from boar and to provide an update on current methods and alternatives to antibiotic use in cold storage.

## 2. Results

### 2.1. Bacterial Presence in Boar Semen

Healthy boars usually do not contain bacteria in their semen. Nonetheless, the preputial diverticulum, skin, and hair of the boar contain several microorganisms. In addition, the collection environment or human intervention can contribute to contamination [2]. Therefore, there are a large number of species of bacteria in boar semen. The percentage of bacteria contaminated semen samples has been described as 32% in 2002 and 2003, 17% in 2005, and 26% in 2006 [2,6,8]. However, this can be even higher (up to 66.7%) in the case of boars [1]. The bacterial and fungal load of fresh boar semen is about 82.41 ± 0.149 × 10^3^ CFU/mL and 0.354 ± 0.140 × 10^3^ CFU/mL after dilution of the ejaculates [9].

Most of the contaminants in semen cultures have been reported to be Gram-negative bacteria belonging to the family Enterobacteriaceae [5,6,10]. Recent studies have evaluated the seminal microbiome of a group of boars *Proteobacteria* (39.1–57.3%), *Firmicutes* (27.5–31.17%), *Actinobacteria* (3.41–14.9%), and *Bacteroidetes* (4.24–5.7%) as the most abundant phyla [11,12]. In addition, the species *Bacillus megaterium*, *Brachybacterium faecium*, and *B. coagulans* were recognized as post-ejaculation contamination from soil, feces, and water sources. On the other hand, a relatively low percentage of *Escherichia coli*, *Clostridium difficile*, *C. perfringens*, *C. botulinum,* and *Mycobacterium tuberculosis* were found in semen [11]. Other studies indicate that species such as *Candida parapsilosis/sakeare* and *Escherichia coli* are found in higher percentages (92% and 81.2%, respectively) [9]. This bacterial contamination can be problematic since they could be pathogenic agents for the sows [11].

MALDI-TOF mass spectrometry and API 20E (Analytical Profile Index) were also used to determine the Gram-negative bacterial species present in porcine seminal samples, where a majority of contaminating bacteria belong to the family *Enterobacteriaceae*, including *Seratia marcescens* (19.56%), *Proteus mirabilis* (15.21%), *E. coli* (10.86%); and to the *Pseudomonaceae* family, including *Ralstonia picketii* (17.39%), *Burkholderia cepacia* (10.86%), *Pseudomonas aeruginosa* (8.69%) and *Pseudomonas fluorescens* (4.34%), respectively [13]. Similar results are reported from Brazil boars’ semen samples, showing 43% contamination with a predominance of Gram-negative bacteria such as *P. aeruginosa*, *P. mirabilis*, *E. coli*, *Kerstersia gyiorum*, *Aerococcus viridans*, *Brevibacterium casei*, *Providencia stuartii*, *Citrobacter koseri*, and *Staphylococcus pasteuri* [14]. *Leptospira* is also present in some samples; however, this is not a pathogen of concern in porcine seminal samples [15].

On the other hand, bacterial diversity changes have been observed in response to seasonal conditions. For example, *Lactobacillus* is highly abundant in samples taken in winter, which are positively associated with sperm quality and reproductive performance. In contrast, *Pseudomonas* is highly abundant in summer samples and is negatively associated with sperm quality and reproductive potential [12]. Metagenomics by high-throughput sequencing results also suggested bacterial community variation from one AI center to another. This is expected due to the different environmental characteristics in which the pigs are kept. In particular, the type of flooring on which the animals are housed might influence bacterial semen contamination [16]. Therefore, providing hygienically impeccable housing conditions will prevent bacterial contamination and preserve spermatic functionality and sanitary quality [17]. Actions such as adequate safeguards against the entry of pathogens from outside, systematic veterinary controls, vaccination, deworming protocols, and pest control are required.

About 60% of the variability in the bacterial contamination profile in semen samples is explained by the hygienic conditions of the different control points in artificial insemination [18]. The process of sample collection has been analyzed step by step to determine the critical points for bacterial contamination. These are the dripping of preputial fluid from the technician’s hand; the collection time over seven minutes; and the presence of long preputial hair [19]. At the laboratory level, sinks or drains show a high rate of bacterial contamination; in fact, multiple isolates of multidrug-resistant bacteria have been isolated from these areas [19]. Elevated bacterial contamination is also found in heating cabinets, ejaculate transfer, manual operating elements, and laboratory surfaces [1]. Moreover, large amounts of resistant bacteria have been reported in the mucosa of the boar’s prepuce [20]. Hygiene management during the collection, maintenance, and insemination processes can significantly reduce bacterial contamination and, therefore, diminish the use of conventional antibiotics [18]. Although this contamination cannot be avoided entirely, it can be contained by appropriate hygiene measures and process optimization [17].

### 2.2. Semen Cold Storage and Bacterial Contamination

AI plays an important role in boar reproduction; 93% of sows are inseminated by artificial insemination, and 99% of all sows are inseminated with extended semen [17,21]. AI is the most widely used reproductive biotechnology in swine. It is almost entirely performed with liquid-preserved semen, as it is used to bridge the time gap between collection and insemination [22]. Preserved extended semen allow for national and international trade of semen doses and increase the efficiency of using an ejaculate, as lower concentrations of spermatozoa are used [22]. Extended semen consists of seminal doses of 50–100 mL at a concentration of 1–3 billion spermatozoa per dose [23]. To obtain a dose, the ejaculate-rich fraction is collected and then diluted, allowing the preparation of multiple required doses [23]. The diluent is an aqueous solution that increases the volume of the ejaculate to the required amount. It helps to reduce the metabolic activity of the spermatozoa, preserves their function, and maintains an adequate level of fertility [23].

There are short-term and long-term extender media; the difference between the two is that the short-term allows liquid preservation for up to three days, while the long-term allows preservation for up to ten days [23]. Usually, the diluted semen is kept at 17 °C for the recommended time. However, microbial growth can occur at this temperature, damaging the quality and functionality of the spermatozoa. Therefore, in recent years, the diluted samples have been preserved at 5 °C [24,25].

Even though bacterial growth can be diminished, strains on contaminated semen samples can still colonize and damage the extended semen. This is why antibiotics are commonly added to each extended semen sample at the time of dilution in order to control or delay the colonization of microorganisms. A recent study evaluated the use of both types of extenders (short- and long-term) in relation to microbial growth. The authors found that total colony counts are not affected by the storage time (3 vs. 7 days); however, Gram-negative counts appear to be higher on day 3 of storage compared to day 7. In addition, the bacterial counts in the short-term extender were higher than those in the long-term extender on the third day of storage [26]. Another study observed that adding an extender supplemented with antibiotic at 17 °C controls bacterial growth for only a few hours. Strains such as *Bacillus* spp., *Pseudomonas* spp., *Staphylococcus* spp., and *Streptococcus* spp. significantly increased their growth after 24 h, failing to adequately maintain the sample quality for five days of storage. Therefore, there is a current necessity in the preservation of semen samples and in the control of their bacterial growth [27].

### 2.3. Consequences of Bacterial Contamination on Boar Semen

**Figure 1:**
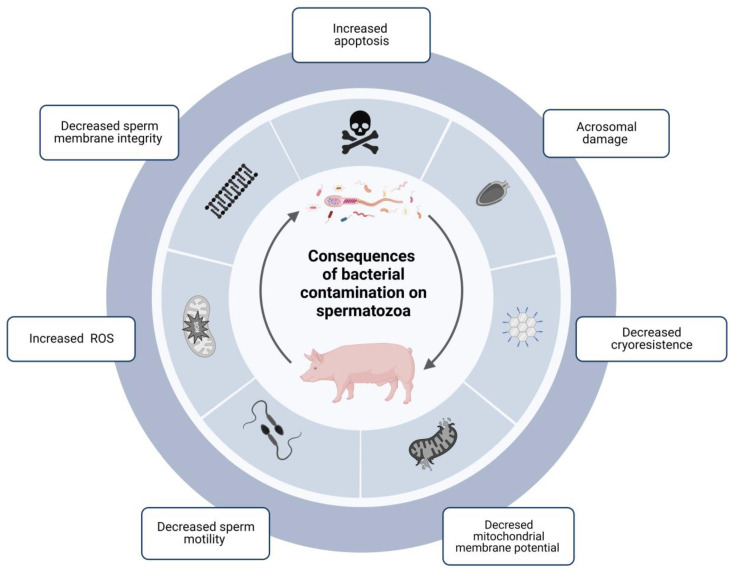
Diagram showing the consequences of the contamination of bacterial communities in boar sperm and how they directly influence sperm quality parameters during cold storage. The presence of bacteria in boar semen has significant consequences on the quality and functionality parameters of spermatozoa: damage to the acrosome, decreased mitochondrial membrane potential, cryoresistance, sperm motility, and integrity of the sperm membrane, and increased ROS and apoptosis. ROS: Reactive oxigen species. Created with BioRender.com.

The degree of bacterial contamination in ejaculates directly influences sperm quality parameters during cold preservation (Figure 1) [28]. Many studies have exemplified the consequences of bacterial growth on spermatozoa, which are summarized in Table 1.

Among the negative consequences recorded for contaminated boar sperm, seminal samples with bacterial counts over 1.4 × 10^4^ CFU/mL showed decreased motility after 108 and 168 h of storage at 16 °C. In addition, the pH decreases from 7.2 to 6.0 between 24 and 168 h of storage in samples with high contamination levels [5,23,29,30,31,32,33]. However, pH changes are species-specific, as *E. coli* and *C. perfringens* infections have been reported to lead to an alkalinization of the seminal medium [34]. In addition, other consequences associated with integrity and function have been described. Some bacteria affect the integrity of the sperm membrane, reducing their viability, damaging the acrosome, and decreasing its cryoresistance [35]. Mitochondrial membrane potential has also been associated with bacterial concentration and storage time [36]. Finally, other studies have demonstrated that bacteria cause agglutination of the cells, preventing the correct mobilization of the spermatozoa [31,33,37,38]. This agglutination is associated with type 1 fimbriae in some bacteria, which bind to glycoprotein receptors on sperm cell surfaces. These fimbriae mediate specific adhesion to the host cell surface. Tail-to-tail, tail-to-head, or head-to-head adhesion occur between spermatozoa [33]. All these consequences have been found to be increased in semen samples stored at 15–17 °C [32] and to a much lesser extent in samples stored at 5 °C; this is why the shelf life of semen doses stored at 15–17 °C is reduced. One study described that the presence of 10^6^ or 10^8^ CFU/mL of *P. aeruginosa* in capacitating media results in low motility of spermatozoa and a decrease in phosphotyrosine—an indicator of sperm capacitation [30]. This result is of great concern since fertilization of oocytes with non- or low-capacitated spermatozoa is inefficient.

Additionally, the adhesion of bacteria to spermatozoa can also occur. Different bacteria, such as *E. coli* and *C. perfringens,* have different adhesion patterns. The proportions of sperm with adhering *E. coli* are higher than those with *C. perfringens* from the fourth day of storage over 5 °C. The adhesion of both bacteria to the spermatozoa surface is non-specific, but it shows a significantly higher adhesion to the middle piece in those infected with *E. coli*, and higher adhesion to the main piece in those infected with *C. perfringens,* causing significantly more damage to the affected area [39]. Additionally, bacterial adhesion increases with higher infective doses and longer storage times [41].

Bacteria, such as *P. mirabilis*, also produce outer membrane vesicles that influence boar sperm function by inducing sperm membrane reconstruction, autophagy, and apoptosis. They affect motility, increase ROS production, alter oocyte binding, increase LC3 and caspase-3 expression, and decrease Bcl2 anti-apoptosis in spermatozoa [40]. In addition, large counts of Gram-negative bacteria produce large amounts of lipopolysaccharides (LPS). LPS can directly bind to the sperm head region, decreasing sperm motility and inducing sperm apoptosis due to its toxicity [42]. Thus, sperm damage is not only directly associated with the bacterial presence on the medium but also with their subproducts. Although functionality and quality are affected, no significant changes in sperm morphology associated with microbial contamination have been described in samples stored for 11 days at 15 °C and 96 h at 37 °C [32].

### 2.4. Antibiotic Use for Preservation of Boar Semen

The addition of antibiotics to the diluent is obligatory by law (EU Directive 90/429/EEC Annex C). Therefore, most commercial sperm media already include antibiotics or those added before semen storage to prevent microbial growth. Several combinations and concentrations have been tested, including 200 μg/mL gentamicin + 200 μg/mL florfenicol, 200 μg/mL gentamicin + 200 μg/mL polymyxin B, 100 μg/mL gentamicin + 100 μg/mL florfenicol and 100 μg/mL gentamicin + and 100 μg/mL polymyxin B. The combination 100 μg/mL gentamicin + 100 μg/mL florfenicol has shown the highest percentage of progressive motility, viability, and mitochondrial membrane potential over ten days. In addition, this combination does not damage DNA integrity, allowing for the desired reproductive performance in terms of the number of pregnant sows and litter size [43]. Sulfanilamide has also been tested for porcine semen storage at 17 °C for 6 days, where concentration of 0.02 g/L is optimal for maintaining the quality parameters. This antibiotic controls the growth of *Staphylococcus* and *Pseudomonas* and enables an increase in litter size and pregnancy rate [44]. In addition, with traditional antibiotics, such as gentamicin, half a dose can reduce the bacterial load to preserve sperm quality and be safe for sow [45]. High concentrations of gentamicin did not have optimal results. In this context, Schulze et al. [46] proposed using an extender without antibiotics and manually supplementing it with the right amount of gentamicin according to the sample volume to ensure its efficiency.

Regarding commercial sperm media, the intrinsic capacity of Androstar^®^ Premium (Minitube) and Beltsville Thawing Solution, BTS^®^ (Pursel and Johnson) is efficient compared to the use of extrinsic antibiotics; therefore, with their use, no extra dose of antibiotics is necessary [45]. On the other hand, using Androstar plus^®^ at 17 °C for 72 h with antibiotics such as gentamicin, aminoglycoside, cephalosporin, lincomycin, and spectinomycin may decrease the total bacterial count. However, gentamicin alone is not sufficiently effective against Gram-positive bacteria. In addition, it was reported that with the combination of three antibiotics, bacterial growth is optimally controlled, showing only a few colonies of *Enterococcus hirae, B. subtilis*, and *Corynebacterium* spp. Therefore, the use of antibiotics should be specific and should not be added randomly in commercial media. Specific antibiotics for semen extension and hygiene standards should preserve semen quality and reduce the indiscriminate use of antibiotics and the antibiotic resistance crisis in the pig industry [47].

The bacterial exposition to different concentrations of antibiotics leads to selection pressure and increases antibiotic resistance emergence. In boar semen preservation, ejaculates are diluted to adjust sperm concentration; therefore, the final antibiotic concentration should depend on the dilution factor of the ejaculate. In some swine AI centers, this method is already being used with successful results [24]. Despite the efforts of some groups to improve and optimize the use of antibiotics, multiple studies have reported antibiotic resistance in semen samples, including *E. coli* against chloramphenicol, *Neisseria meningitides* against spectinomycin, and *S. aureus* against linezolid [48]. Worryingly, Costinar et al. [13] observed that 56.52% of isolates were resistant to gentamicin and 58.69% to penicillin, demonstrating that the indiscriminate use of antibiotics stimulates antibiotic resistance [13]. Table 2 shows the various studies on the use of antibiotics and other alternatives that have been tested for the control of bacterial growth in chilled boar semen samples over the last few years.

### 2.5. Alternatives to Prevent Bacterial Contamination in Semen Storage

In recent years, there have been increasing efforts to develop alternative methodologies or additives to avoid using conventional antibiotics (Table 2). A potential additive must meet the following criteria: (a) a broad spectrum of antimicrobial action, (b) no toxicity to sperm, (c) no interference with fertility, (d) high stability, (e) high activity at ordinary semen storage temperatures, (f) low potential to cause bacterial resistance, (g) ease of application, and (h) economic feasibility [63,64].

An action proposed to tackle microbial growth is the strategic management of the sample collection process to reduce contamination with microorganisms. Ciornei et al. [9] recommend a modification of the protocol for sperm collection, carrying out a hygiene protocol, and the biosecurity of sperm collection, which reduces bacterial contamination in raw sperm by 49.85%. This protocol involves applying lightly decontaminating substances (e.g., Misoseptol) 15 min before the collection, providing the operator with two latex gloves and disinfecting the foreskin region, emptying the foreskin diverticulum of secretions and urine [9].

Another proposed strategy to reduce contamination is the hypothermic preservation of boar semen. Jäkel et al. [65] described the semen quality of boar samples cooled to 5 °C and 17 °C. They found a decrease in viable spermatozoa with low membrane fluidity up to 72 h in sperm stored at 5 °C compared to those stored at 17 °C, but the same differences are not observed after 144 h. Similar results were observed in other studies evaluating boar semen samples stored for 120 h at 5, 10, and 17 °C, with or without antibiotics added to the preservation medium. Total motility is more than 75% on semen stored at 5 and 10 °C. However, total, and progressive motility increase with higher storage temperatures in doses containing antibiotics. Significantly lower motility is observed at 5 °C compared to higher temperatures in doses without antibiotics [25]. Sperm preservation in AndroStar Premium diluent at 5 °C maintains high motility, membrane integrity, and a low DNA fragmentation rate for 72 h. In addition, there is a positive kinetic response to the bicarbonate capacitation stimulus during 180 min incubation. In addition, competitive binding of sperm to the oviduct and fertility rates shows no differences with the use of semen preserved at 5 or 17 °C [66]. Although a percentage of semen quality is negatively affected by low temperatures, storage of boar doses for extended periods at 5 °C is possible, as in vitro sperm viability was maintained for up to 5 days, meeting semen quality requirements for use in artificial insemination.

As expected, bacterial growth is diminished at lower temperatures; then, in semen stored at 5 °C, bacterial counts are lower than in semen stored at 17 °C, staying below 10^3^ CFU/mL and showing a similar bacterial spectrum to raw semen [65,66]. This effect can be observed in samples extended without antibiotics; however, no effect of storage temperature is observed in semen doses extended with antibiotics. Other studies proposed a cooling-rate frame for boar semen at 5 °C in an antibiotic-free extender. Sperm motility, membrane integrity, membrane fluidity, mitochondrial membrane potential, and response to the bicarbonate capacitation stimulus were optimally maintained for 144 h at 5 °C when semen is initially cooled in a cooling rate frame ranging from 0.01 to 0.09 °C/min in the 30 to 25 °C temperature zone, followed by 0.02 to 0.06 °C/min at 10 °C and 0.01 to 0.02 °C/min to the final storage temperature. Slower cooling might result in more bacterial growth; for example, a holding time of 6 h at 22 °C resulted in an increase of *E. coli* from 0.5 × 10^3^ to 2.4 × 10^3^ CFU/mL after 6 h of storage in a diluent inoculated without sperm [67]. Finally, fertility is high and does not differ between the groups of sows inseminated with semen stored without antibiotics at 5 °C and at 17 °C with antibiotics [65].

Alternatively, it is possible to use the physical separation of spermatozoa from bacteria, thus avoiding the use of antibiotics. To this end, two low-density Porcicoll (P20 and P30) has been evaluated, and a considerable reduction or complete elimination of some bacteria by both colloids, without affecting sperm quality, was observed. Furthermore, the recovery rates are 86% for P20 and 81% for P30, presenting a very efficient alternative to the use of antibiotics [68]. In addition, spermatozoa can be separated from bacteria in semen via single-layer centrifugation (SLC). SLC plus a swim-up stage has been proven to remove 99% of pathogens from semen samples [69]. Using the colloid AndrocollTM-P is possible to eliminate between 60–40% of the bacteria. However, there are also more difficult microorganisms to remove; for example, flagellated motile bacteria are more challenging to eliminate than non-flagellated ones [70]. Martínez-Pastor et al. [68] evaluated SLC on modified low-density Porcicoll. A sterile inner tube is included inside some 50 mL centrifuge tubes to facilitate harvesting of the sperm pellet. Both methodologies significantly decrease the count of bacteria compared to the control, presenting a higher percentage of motile and fast spermatozoa [71].

### 2.6. Use of Supplements and Other Antimicrobial Molecules

**Figure 2:**
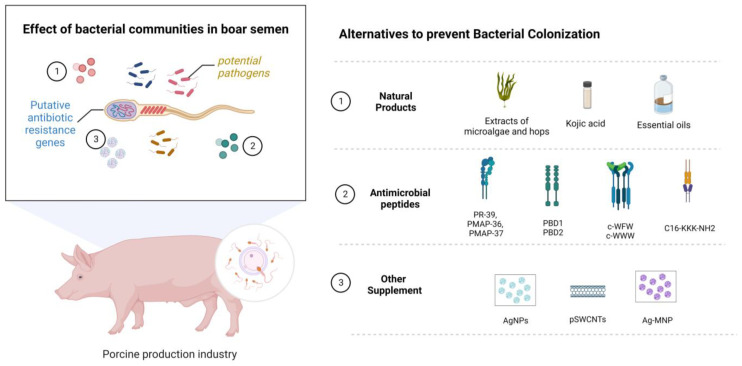
Boar semen contain a large bacterial community, including potential pathogens and putative antibiotic resistance genes. For this reason, different supplements have been evaluated that could contribute to decreasing the bacterial load in semen and maintaining quality parameters under freezing conditions and subsequent use in the industry. Within this group, we find natural molecules, antimicrobial peptides, and other supplements. Proline-arginine-rich antimicrobial peptides PR-39 (PR-39), porcine myeloid antimicrobial peptides 36 (PMAP-36), and 37 (PMAP-37), synthetic cyclic hexapeptides (c-WFW, c-WWW), pegylated single-walled carbon nanotubes (pSWCNTs), and magnetic silver nanocomposites (Ag-MNP). Created with BioRender.com.

During the last few years, various additives have been used in the search for the control of bacterial contamination without the addition of antibiotics (Table 2 and Figure 2). Hensel et al. [54] examined bioactive extracts from microalgae and hops to evaluate their effect on the quality and bacterial load of porcine semen preserved at 5 °C. In general, bacterial growth was not completely inhibited by the bioactive extracts; however, all extracts showed an antimicrobial effect on specific bacteria, including Gram-positive bacteria (*Trueperella pyogenes* and *Streptococcus porcinus*) and Gram-negative bacterial species (*Alcaligenes faecalis*, *P. aeruginosa*, *Ralstonia* sp., *Pasteurella* sp., *Proteus* sp., *Providencia stuartii* and *E. coli*). Another natural compound tested was kojic acid (0.04 g/L) on spermatozoa stored at 17 °C, which improves sperm quality and capacitation parameters. This treatment increases total antioxidant capacity and sperm binding, cleavage, and blastocyst rates, and reduces bacterial concentrations. Finally, it was found that kojic acid increases the expression of the antioxidant proteins SOD1, SOD2, and CAT, and the anti-apoptosis protein Bcl 2, and decreases the expression of the apoptosis-related proteins caspase 3 and Bax in sperm, making it a good antagonist of sperm apoptosis. Therefore, this compound is postulated as an alternative for controlling bacterial contamination and preserving sperm quality and functionality [51]. In addition, different essential oils have been tested on spermatozoa to assess their toxicity before being considered suitable substitutes for standard antibiotics. The potential antimicrobial effects of *Melaleuca alternifolia* and *Rosmarinus officinalis* essential oils were evaluated. As a result, a concentration of 0.4 mg/mL of both oils can produce similar effects to ampicillin, which is a suitable alternative to this antibiotic [50]. A recent study determined that adding 0.5 and 0.6% buckwheat honey significantly improved sperm motility, acrosome integrity, plasma membrane integrity, inhibition of opportunistic bacterial growth, and altered microbial composition at the storage end. In addition, the levels of total antioxidant capacity, catalase, and superoxide dismutase enzymes are higher with buckwheat honey [55].

Another additive strategy is the use of antimicrobial peptides (AMP). Antimicrobial peptides possess three mechanisms against bacteria: (a) Positively charged cationic AMPs interact with the negative charge of the cell membrane, generating conformational changes. (b) Following the binding of antimicrobial peptides to the cell membrane, different mechanisms of action are generated, forming different types of pores: annular or toroidal pore model, ring pore or toroidal pore model, barrel pore model, and carpet pore model. (c) AMPs can interact directly with DNA, RNA, ribosomes, and perturbed chaperone proteins, disrupting processes such as cell division, replication, and nucleic acid synthesis [72]. AMP has become one of the most promising alternatives to using antibiotics in semen extender’s formulations to overcome the increasing bacterial resistance to antibiotics. However, AMP may impair boar sperm quality. To test this, the effects of three AMPs were evaluated during cold storage of semen, including the proline-arginine-rich antimicrobial peptide PR-39 (PR-39), porcine myeloid antimicrobial peptides 36 (PMAP-36), and 37 (PMAP-37). The results showed that all tested AMPs decrease sperm motility, while PMAP-37 is more effective in maintaining sperm viability than antibiotic controls. In addition, PR-39 and PMAP-37 at 3 μM effectively control the bacterial load, keeping it low during the ten days of cold storage [58]. Other AMPs, such as the antimicrobial peptides porcine beta defensins-1 (PBD1) and 2 (PBD2), also showed inhibition of bacterial growth—although less than antibiotic control—preserve sperm viability and motility equivalent to the antibiotic control in preservation at 17 °C for ten days. Therefore, their use is postulated as an antibiotic supplement, as they do not entirely control bacterial growth [60].

The cationic antimicrobial peptides are a new class of antimicrobial additives for boar semen preservation. The use of synthetic cyclic hexapeptides (c-WFW, c-WWW) has been found to partially stimulate the progressive linear movement of spermatozoa. Furthermore, at low concentrations of 4 mM c-WFW and 2 mM c-WWW, the sperm quality is comparable to that of the standard diluent throughout storage. In addition, boar semen supplemented with C-WFW allow regular fertility rates after AI. Therefore, the authors suggest that this may be the first approach to test synthetic cyclic hexapeptides’ efficiency as antimicrobials during boar spermatozoa cooling [56]. In response to this need, the same synthetic cationic antimicrobial peptides c-WWW, c-WFW, and a helical amide analog of magainin II (MK5E) were evaluated for Gram-positive and Gram-negative bacterial contamination in boar semen. The three cationic antimicrobial peptides showed activity against most bacteria but not against *Proteus* spp. and *Staphylococcus aureus* ATCC 29213 (only the case of MK5E). Furthermore, c-WWW and c-WFW are effective against bacterial growth in boar semen preserved in situ, especially when combined with a small amount of gentamicin. Therefore, cationic peptides are postulated as an alternative to decrease the use of antibiotics, although they cannot replace them entirely [57]. Microbial lipopeptides have also been tested. C16-KKK-NH2 and C16-KKK-NH2 do not adversely affect sperm quality. In addition, both lipopeptides led to an overall decrease in CFU counts, with a decrease in Gram-positive bacilli by 40% compared to the control and 60% Gram-negative species. In addition, some lipopeptides reveal activity against bacteria of concern in AI, such as *T. pyogenes*, *Alcaligenes faecalis*, *P. aeruginosa*, *Pasteurella* sp., *P. stuartii*, *E. coli,* and *S. porcinus* [73]. Finally, iodine methionine (IM), which participates in the production and activation of metabolic enzymes, is a new type of amino acid chelate. A study showed that 80 μM IM improve motility, plasma membrane integrity, and acrosome integrity, resulting in a larger litter size than the control group. In addition, IM inhibits the proliferation of the phylum Proteobacteria, the genre *Staphylococcus*, and *Pseudomonas* [59].

There have been significant advances in nanotechnology. Pegylated single-walled carbon nanotubes (pSWCNTs) and magnetic silver nanocomposites (Ag-MNP) have antibiotic activity against *E. coli* and *Salmonella*, delaying bacterial growth and changing protein expression [74]. Silver nanoparticles (AgNPs) 10–20 nm in size have shown a biocidal effect on bacterial microorganisms without toxicity to certain mammalian cells. Specifically, in chilled porcine spermatozoa, it has been observed to preserve viability, mitochondrial metabolism, membrane integrity, morphology, and no changes associated with capacitation or acrosomal reaction at 4 mM concentrations. It also decreases the proliferation of *S. aureus* [62]. In addition, this dose is not cytotoxic to spermatozoa, as it allows sperm viability to be maintained. Thus, they could be used to prevent bacterial growth in samples intended for storing and conserving porcine germplasm without toxic effects [61]. Nanotechnology is still a developing area; hence, advances that could achieve better results are foreseen to optimize and validate its use in sperm preservation.

## 3. Future Prospects and Challenges

Boar semen contains a bacterial community that includes potential pathogens with putative antibiotic resistance genes that may affect boar reproductive performance. Currently, bacterial contamination in semen is a significant concern within the “One Health” concept, as commercial semen doses in the livestock industry contribute to spreading pathogens and antibiotic resistance genes [11]. There is a misguided view in the pursuit of total elimination of bacterial growth by some breeding organizations, as it has been observed that thresholds for aerobic mesophilic bacteria between ×10^3^ and ×10^7^ CFU/mL can be allowed, as they do not produce adverse effects on sperm quality or fertility. That is important, as it implies a change in the industry’s view of the problem, which would lower the standards so that such high use of antibiotics would not be required [22]. The animal production industry has a significant challenge: to increase production efficiency but, at the same time, reduce the indiscriminate use of antibiotics. Today, there are still countless bad practices and regulations that insist on their use. The free, preventive, and unsupervised use of antibiotics has led to years of bacterial evolution toward antibiotic resistance, where, unfortunately, current antibiotics have practically no effect whatsoever. Therefore, current advances have allowed us to determine that we must use three crucial strategies to tackle the problem: first, to avoid the excessive preventive use of antibiotics; second, to establish a system of good hygiene and cleaning practices for sample collection; and finally, to continue the search for new molecules with antibiotic activity, which will allow us not only to deal with pathogens in semen samples but also with all types of human and animal pathogens.

## Figures and Tables

**Table 1:** Consequences reported in porcine spermatozoa of bacterial growth during cold preservation.

**Authors**	**Species**	**Total Motility**	**Membrane Integrity**	**Acrosome Integrity**	**Mitochondrial Membrane Potential**	**Capacitation Ability**	**Ros Levels**	**Lipid Peroxidation**
Bussalleu et al., 2011 [29]	enterotoxigenic *Escherichia coli (ETEC)* and verotoxigenic *Escherichia coli (VTEC)*	↓	↓	-	-	-	-	-
Úbeda et al., 2013 [10]	*Klebsiella oxytoca, Serratia marcenses*, *Proteus mirabilis* and *Morganella morganii*	↓	-	-	-	-	-	-
Sepúlveda et al., 2013 [32]	*Clostridium perfringens*	↓	↓	-	-	-	-	-
Prieto-Martínez et al., 2014 [33]	*Enterobacter cloacae*	↓	↓	-	-	-	-	-
Sepúlveda et al., 2014 [31]	*Pseudomonas aeruginosa*	↓	↓	↓	-	-	-	-
Sepúlveda et al., 2016 [30]	*Pseudomonas aeruginosa*	↓	-	-	-	↓	-	-
Pinart et al., 2017 [34]	*Escherichia coli* and *Clostridium perfringens*	↓	↓	-	-	-	-	-
Bonet et al., 2018 [39]	*Escherichia coli* and *Clostridium perfringens*	-	↓	↓	-	-	-	-
Ďuračka and Tvrda 2018 [36]	*Escherichia coli, Pseudomonas aeruginosa,* and *Staphylococcus aureus*	↓	-	-	↓	-	↑	↑
Ďuračka and Tvrda 2018 [36]	*Rothia nasimurium, Acinetobacter lwoffii,* and *Staphylococcus simulans*	ND	ND	ND	ND	ND	↑	↑
Gao et al., 2018 [40]	*Proteus mirabilis*	↓	-	-	-	-	↑	-
Schulze et al., 2018 [38]	*Lactobacillus*	↓	↓	-	↓	-	-	-
Delgado-Bermúdez et al., 2020 [41]	*Proteus vulgaris*	-	↓	↓	-	-	-	-

ROS: Reactive oxygen species; ND: No differences.

**Table 2:** Studies on the use of antibiotics or alternative molecules in porcine sperm storage in recent years.

	Authors	Added Compound	Recommended Concentrations
Antibiotics	Bryla et al., 2015 [43]	Gentamicin, florfenicol and polymyxin B.	Combination of 100 μg/mL gentamicin and 100 μg/mL florfenicol
	Schulze et al., 2017 [46]	Gentamicin	512 mg/L
	Feng et al., 2019 [44]	Sulfanilamide.	0.02 g/L
	Shaoyong et al., 2019 [49]	ε-Polylysine	0.16 g/L
	Luther et al., 2021 [45]	Androstar^®^ Premium, Beltsville Thawing Solution, BTS^®^, Gentamicin, Apramycin, Ampicillin	Gentamicin 0.1 mg/mL, Apramycin 0.125 mg/mL and Ampicillin 0.125 mg/mL
	Tvrdá et al., 2021 [47]	Gentamicin, aminoglycoside, cephalosporin, lincomycin, spectinomycin	-
Natural Compounds	Elmi et al., 2019 [50]	*Melaleuca alternifolia* and *Rosmarinus officinalis*	0.4 mg/mL
	Shaoyong et al., 2019 [51]	Kojic acid	0.04 g/L
	Lustykova et al., 2020 [52]	Gallic acid	0.008 mol/L
	Manpoong et al., 2020 [53]	Turmeric and KMnO_4_	Turmeric 0.5 mM and KMnO_4_ 10 μM
	Hensel et al., 2021 [54]	Extracts from microalgae and hops	2 μg/mL
	Lan et al., 2021 [55]	Buckwheat honey	0.5%
Antimicrobial Peptides	Schulze et al., 2014 [56]	c-WFW and c-WWW	4 μM
	Speck et al., 2014 [57]	c-WWW, c-WFW, and MK5E	c-WWW 2 μM, c-WFW 4 μM and MK5E 1 μM
	Bussalleu et al., 2017 [58]	PR-39, PMAP-36, and PMAP-37	10 μM
	Fang et al., 2017 [59]	Iodine methionine	80 μM
	Puig-Timonet et al., 2018 [60]	PBD1 and PBD2	3 μM
	Hensel et al., 2021 [54]	C16-KKK-NH_2_ and C16-KKKK-NH_2_	8 μg/mL
Nanoparticles	López-Pérez et al., 2017 [61]	AgNPs	4 mM
	Pérez-Durán et al., 2020 [62]	AgNPs	20 mg/mL

PR-39: Proline-arginine-rich antimicrobial peptide 39, PMAP-36: Porcine myeloid antimicrobial peptides 36, PMAP-37: Porcine myeloid antimicrobial peptides 37, PBD1: Peptides porcine beta defensins-1, PBD2: Peptides porcine beta defensins-2, c-WFW: synthetic cyclic hexapeptide c-WFW, c-WWW: synthetic cyclic hexapeptide c-WWW, MK5E: Helical amide analog of magainin II, C16-KKK-NH2 and C16-KKK-NH2: Microbial lipopeptides, AgNPs: Silver nanoparticles, pSWCNTs: Pegylated single-walled carbon nanotubes, Ag-MNP: magnetic silver nanocomposites.

## Data Availability

Not applicable.
